# Does IQ affect the functional brain network involved in pseudoword reading in students with reading disability? A magnetoencephalography study

**DOI:** 10.3389/fnhum.2013.00932

**Published:** 2014-01-08

**Authors:** Panagiotis G. Simos, Roozbeh Rezaie, Andrew C. Papanicolaou, Jack M. Fletcher

**Affiliations:** ^1^Department of Psychiatry, School of Medicine, University of CreteHerakleion, Crete, Greece; ^2^Department of Pediatrics, Division of Clinical Neurosciences, College of Medicine, University of Tennessee Health Science CenterMemphis, TN, USA; ^3^Neuroscience Institute–Le Bonheur Children’s HospitalMemphis, TN, USA; ^4^Department of Psychology, University of HoustonHouston, TX, USA

**Keywords:** magnetoencephalography, functional brain imaging, reading, dyslexia, intelligence

## Abstract

The study examined whether individual differences in performance and verbal IQ affect the profiles of reading-related regional brain activation in 127 students experiencing reading difficulties and typical readers. Using magnetoencephalography in a pseudoword read-aloud task, we compared brain activation profiles of students experiencing word-level reading difficulties who did (*n* = 29) or did not (*n* = 36) meet the IQ-reading achievement discrepancy criterion. Typical readers assigned to a lower-IQ (*n* = 18) or a higher IQ (*n* = 44) subgroup served as controls. Minimum norm estimates of regional cortical activity revealed that the degree of hypoactivation in the left superior temporal and supramarginal gyri in both RD subgroups was not affected by IQ. Moreover, IQ did not moderate the positive association between degree of activation in the left fusiform gyrus and phonological decoding ability. We did find, however, that the hypoactivation of the left pars opercularis in RD was restricted to lower-IQ participants. In accordance with previous morphometric and fMRI studies, degree of activity in inferior frontal, and inferior parietal regions correlated with IQ across reading ability subgroups. Results are consistent with current views questioning the relevance of IQ-discrepancy criteria in the diagnosis of dyslexia.

## INTRODUCTION

An enduring issue concerning both the definition and diagnosis of developmental reading disability (RD) rests in the use of measures of general cognitive ability (operationalized as IQ scores). Traditional definitions of RD not only require general intellectual functioning within the normal range but often set a minimum degree of discrepancy between reading achievement standard scores and IQ in order for a diagnosis of RD to be made (Fletcher et al., 2007). In earlier studies, poor readers who read at levels consistent with their IQ scores (low achievers) differed from poor readers who read well below their IQ levels (IQ-discrepant) on a variety of attributes, including prognosis, severity of reading difficulties, gender, and some cognitive abilities (Rutter and Yule, 1975). Subsequent research, however, has not replicated these findings, suggesting that these earlier findings reflected inclusion of children with intellectual disabilities and brain injury. More recent meta-analyses have reported little difference between IQ-discrepant and low achieving poor readers on reading skills and cognitive abilities closely related to reading (phonological awareness, rapid naming, working memory, and vocabulary; Hoskyn and Swanson, 2000; Stuebing et al., 2002; see also Fletcher et al., 1994; Stanovich and Siegel, 1994). Further evidence suggested that the two subgroups of RD children show comparable response to intervention and overall prognosis (Francis et al., 1996; Vellutino et al., 2000; Stuebing et al., 2009).

Despite these findings, it is common in neuroimaging studies to select participants based on either IQ-discrepancy criteria or to use relatively high IQ cut-offs. Such an approach is supported by evidence for small differences in the heritability of reading skills in poor readers with higher and lower IQ scores (Wadsworth et al., 2010). In this line of research, assessments of large twin samples have suggested that RD in children with higher IQ scores has a stronger genetic etiology. These practices and findings would suggest differences in the functional organization of the brain networks engaged during reading tasks between IQ-discrepant and low achieving poor readers.

Numerous neuroimaging studies using fMRI and magnetoencephalography have documented reduced activation among students with RD, as compared to typical readers. These differences are most apparent in left hemisphere regions purportedly involved in phonological processes, namely in the posterior portion of the superior temporal gyrus, and the supramarginal gyrus (Temple et al., 2001; Shaywitz et al., 2002; Simos et al., 2007, 2011), often extending into the adjacent angular gyrus (Shaywitz et al., 1998; Temple et al., 2001; Simos et al., 2007). Several studies have also reported reduced activation in left hemisphere ventral and/or lateral occipitotemporal regions believed to be involved in orthographic/graphemic processing (Shaywitz et al., 2002; McCrory et al., 2005; Cao et al., 2006; Hoeft et al., 2007a; van der Mark et al., 2009). Reports of changes in inferior frontal activity during reading in RD are more variable (e.g., Shaywitz et al., 2002; Cao et al., 2006; Hoeft et al., 2007a).

The research question addressed in the current study is whether variability in general cognitive ability as indexed by performance and verbal IQ makes a difference in neuroimaging studies as it seems to make in behavioral genetic studies. To our knowledge there has been a single attempt to assess the neurophysiological plausibility of the IQ-discrepancy criterion: Tanaka et al. (2011) reported fMRI data obtained during performance of a word-rhyme task from 69 struggling readers (identified by a score below the 25th percentile on a word reading accuracy test) and 62 typical readers. The RD sample was further subdivided into a high- and a low-IQ group as defined by a cutoff standard score of 90 points on the Peabody Picture Vocabulary Test. This study did not explicitly manipulate the IQ-discrepancy criterion. However, although both groups of RD students scored, on average, lower on this measure of verbal IQ than the group of typical students, the majority of students in the high-IQ group met the IQ-discrepancy criterion, whereas the majority of students in the low-IQ group did not. Analyses focusing on three ROIs per hemisphere revealed that both RD groups showed reduced hemodynamic activation in the left ventral occipitotemporal region (fusiform gyrus) and the left inferior parietal lobule (mainly the supramarginal gyrus). Complementary whole brain analyses showed that none of the activation sites that distinguished typical from poor readers differed in the degree of hemodynamic activity between the two groups of poor readers.

The results of the large-scale study by Tanaka et al. (2011) are important in demonstrating that the functional organization of the brain network involved in phonological processing of print does not vary as a function of verbal IQ in poor readers. These findings leave, however, the possibility open that poor readers may engage additional regions depending on their general cognitive ability. Although regions such as the dorsolateral prefrontal cortex, the superior parietal lobule, and the anterior cingulate are not generally considered parts of the brain network *specialized* for reading, there is increasing evidence implicating these regions in IQ-related functions, including spatial cognition, executive functions, and attention. For instance, the degree of task-related hemodynamic activity in the dorsolateral prefrontal cortex is affected by IQ (Perfetti et al., 2009), whereas the degree of age-related increase in cortical thickness in superior frontal cortices is significantly higher among high-IQ persons over the age range covered by the present study (Shaw et al., 2006). Concurrent recruitment of dorsolateral prefrontal and posterior superior parietal cortices has been linked to the ability to solve non-verbal reasoning problems similar to those included in the majority of common IQ tests (Kroger et al., 2002; Choi et al., 2008). Further, the importance of posterior superior parietal, along with superior, middle, and inferior prefrontal, regions for general intelligence and executive function is supported by recent voxel-based lesion studies (Gläscher et al., 2010; Barbey et al., 2012). The anterior cingulate gyrus has been implicated in attention especially under increasing task difficulty conditions-as when reading rapidly presented, unfamiliar printed stimuli by RD students (Cattinelli et al., 2013). An additional visual area (cuneus) was also examined in view of its suggested role in the control of visual attention (Vanni et al., 2001; Simpson et al., 2011) in tasks that include reading (Osipowicz et al., 2011).

The primary goal of the present study was to extend these fMRI findings in three directions. First, we directly compared two groups of RD students who either met or did not meet the IQ-discrepancy criterion on non-verbal IQ, as well as verbal IQ. Second, we assessed the potential moderating role of IQ in determining reading-related activity, not only among struggling readers but also among typical readers. Third, we expanded the research question from hemodynamic to neurophysiological measures occurring in real time, using magnetoencephalography. We compared brain activation profiles of students experiencing word-level reading difficulties (*n* = 65) who either met the IQ-reading achievement discrepancy criterion (*n* = 29) or did not meet this criterion (*n* = 36) while performing a pseudoword read-aloud task. Two groups of typical readers served as comparisons: students in the lower-IQ group served as controls for the non-discrepant RD subgroup (*n* = 18), whereas students in the higher-IQ group (*n* = 44) served as controls for the IQ-discrepant RD subgroup. We hypothesized that the children with higher IQ scores, whether verbal or performance, would show increased activation in regions outside the reading network related to language and spatial cognition, but not in reading-related areas.

## MATERIALS AND METHODS

### PARTICIPANTS

Magnetoencephalography data were obtained from 127 students aged 6.5–14.3 years. The sample included 65 children experiencing reading difficulties (RD group), as indicated by scores below the 25th percentile (standard score of 90) on the Basic Reading composite [average of Word Attack (WA) and Letter-Word Identification (LWID)] subtest scores of the Woodcock–Johnson Tests of Achievement-III [W-J III], Woodcock et al., 2001). A group of 62 children who had never experienced reading difficulties and had scores >92 on the basic reading composite index served as controls. **Table 1** displays demographic and psychoeducational information for each of the four groups of participants, which were comparable on age, ethnicity, handedness, and Performance IQ (PIQ). All participants had full scale (FSIQ) scores >77 on the Wechsler Abbreviated Scale of Intelligence (Wechsler, 1999) and no history or current indications of ADHD as indicated by *T* scores <55 on the attention problems scale of the parent form of the Child Behavior Checklist (CBCL; Achenbach, 1991) or a mean score lower than 1.67 on the inattention and hyperactivity-impulsivity scales of the parent-completed Swanson, Nolan, Achenbach, Pelham questionnaire (SNAP-IV; Swanson, 1992) indicating low risk for ADHD (Chen et al., 1994). Fourteen low-achieving and nine typically achieving students were excluded from the current cohort for not meeting the aforementioned ADHD criteria.

**Table 1:** Demographic data and performance on standardized tests for each group of participants (mean and SD in parentheses).

	RD		Typical readers	
	IQ-discrepant (higher IQ; *n* = 29)	Non-discrepant (lower IQ; *n* = 36)	Lower IQ (*n* = 18)	Higher IQ (*n* = 44)
Gender (m/f)	22/7	25/11	13/5	26/18
Age (mo)	135.41 (20.52)	140.54 (26.23)	107.3 (33.3)	116.33 (26.19)
WA	81.03 (4.37)	82.58 (6.79)	110.44 (9.81)	108.17 (9.67)
LWID	74.45 (8.16)	75.33 (11.97)	108.38 (11.01)	107.48 (11.97)
Spelling	73.68 (16.56)	75.52 (11.19)	113.62 (13.41)	110.93 (12.65)
VIQ	109.48 (12.31)	90.37 (8.21)	89.06 (12.83)	113.42 (14.90)
PIQ	105.09 (11.38)	87.03 (12.01)	92.44 (10.60)	106.89 (14.26)
% correct	51.37 (17.84)	56.67 (16.58)	93.09 (2.87)	88.51 (11.32)

*Woodcock–Johnson Tests of Achievement-III subtests: Word Attack (WA), Letter-Word Identification (LWID), Spelling. VIQ and PIQ indices derived from the Wechsler Abbreviated Scale of Intelligence. % correct: Percent correct pseudowords read aloud on the activation task in the scanner.*

Following Tanaka et al. (2011) and Wadsworth et al. (2010), each group was further divided into two subgroups based on FSIQ. RD students in the higher-IQ, discrepant group scored at least one standard deviation higher on FSIQ than on the WJ Reading Composite (range: 96–123 points). In all cases, students in the lower-IQ, non-discrepant group had FSIQ scores within one SD from their reading achievement standard scores (range: 77–96 points). A median split (corresponding to an FSIQ score of 95) was set to assign typical readers into a higher- (range: 95–131) and a lower-IQ group (range: 77–94 points).

The two RD groups did not differ on age, on any of the standardized reading measures or on in-scanner performance on the activation task (*p* > 0.19). By definition the IQ-discrepant RD group had higher average VIQ and PIQ than the non-discrepant RD group, *F*(1,64) = 56.11, *p* = 0.0001 and *F*(1,64) = 19.68, *p* = 0.0001, respectively. The two groups of typical readers were also comparable on age, in-scanner performance and standardized reading measures (*p* > 0.09). By definition, the Higher-IQ subgroup of typical readers had higher VIQ and PIQ score than the Lower IQ subgroup of typical readers, *F*(1,61) = 25.82, *p* = 0.0001 and *F*(1,61) = 10.54, *p* = 0.001, respectively.

Across IQ subgroups, typical readers were older than their RD peers [main effect of RD Group: *F*(1,126) = 34.22, *p* = 0.0001], and scored higher (main effects of RD Group) on WA, *F*(1,126) = 439.12, *p* = 0.0001, LWID, *F*(1,126) = 239.71, *p* = 0.0001, Spelling, *F*(1,126) = 254.09, *p* = 0.0001, and in-scanner performance: *F*(1,126) = 87.32, *p* = 0.0001. By design the IQ-discrepant RD subgroup and the High-IQ typical reading group were comparable on PIQ and VIQ (*p* > 0.07), as were the non-discrepant RD subgroup and the Lower IQ subgroup of typical readers (*p* > 0.1).

### PROCEDURE

#### Task

Each participant was tested on a pseudoword read-aloud task involving three-letter pronounceable non-words (e.g., *lan*), subtending 2.0° of visual angle. For each task 100 stimuli were presented randomly arranged in four blocks of 25 items each. Stimuli were presented for 1 s, one at a time (with a randomly varied interstimulus interval of 3–4 s), through a Sony LCD projector (Model VPL-PX21) on a back-projection screen located approximately 60 cm in front of the participant. Participants were instructed to name read aloud each letter string immediately after it had disappeared from the screen. Prior to each scan children were asked to practice this response strategy while magnetic activity was monitored online to ensure that movement artifacts associated with articulation systematically occurred only *after* the end of the recording epoch. Epochs containing such movement artifacts (when, occasionally, verbal responses were produced earlier than instructed) were not included in the data analyses.

#### Imaging procedures

Magnetoencephalography recordings were obtained with a whole-head neuromagnetometer array (4-D Neuroimaging, Magnes WH3600), that consisted of 248 first-order axial gradiometer coils, housed in a magnetically shielded chamber and arranged to cover the entire head surface. The magnetic flux measurements were digitized at 250 Hz, filtered with a bandpass filter between 0.1 and 20 Hz and subjected to baseline adjustment (using the 150 ms prestimulus recording) and to a noise reduction algorithm that is part of the 4D-Neuroimaging software. The single-trial event-related field segments (ERFs) in response to 60–80 stimulus presentations, were averaged after excluding those containing eye movement or other myogenic or mechanical artifacts.

To identify the intracranial origin of ERFs, the magnetic flux distribution recorded simultaneously over the entire head surface at successive points (4 ms apart) was analyzed using a minimum norm model to obtain estimates of the time-varying strength of intracranial currents (Hamalainen M. MNE Software Users Guide. Version 2.5. Charlestown, MA: 2006). This method affords greater spatial resolution and allows detection of simultaneous magnetic sources distributed along the entire cortical surface. The model assumes a continuous distribution of current along the cortical surface which has some minimum norm (Hämäläinen and Ilmoniemi, 1994). Estimated current sources were anatomically constrained by an MRI-derived surface model of each participant’s brain (T1-weighted: TR 13.6 ms; TE 4.8 ms; recording matrix 256 × 256 pixels, 1 excitation, 240 mm field of view, and 1.4 mm slice thickness), obtained on a Philips 3 T scanner with SENSE (Sensitivity Encoding) technology.

This surface model was generated by a fully automated cortical surface reconstruction procedure using FreeSurfer software (Dale et al., 1999) for producing a detailed geometric description (regular tessellation of the cortical surface consisting of equilateral triangles known as vertices) of the gray-white matter boundary of the neocortical mantle and the mesial temporal lobe. Each hemisphere consisted of approximately 150,000 vertices (depending on each subject’s cortical surface area). For estimating current sources, the MNE software requires the Freesurfer-derived cortical surface reconstruction for defining the boundaries of a solution source space. A grid-spacing of 7 mm was used to construct icosahedrons to decimate the number of vertices from 150,000 to approximately 3,000 per hemisphere. Additionally, the MNE software was used to construct a single compartment boundary element model using triangular tessellations to model each vertex as a potential current dipole perpendicular to the cortical surface during the forward calculations. The inverse solution was subsequently reduced to obtain an estimate of the scalar distribution of dipole strength across current sources within orientation-specific cortical patches of vertices (Dale et al., 1999). Co-registration of each MEG dataset with its corresponding MRI dataset was performed using an automated co-registration routine within MNE which aligns digitization points in the MEG headshape file with the fiducial points demarcated on the outer skin surface reconstruction of the MRI.

As in our previous report from this cohort (Simos et al., 2011) activity was examined in the following set of ROIs (separately in each hemisphere): superior temporal gyrus (STG; BA 22); supramarginal gyrus (SMG; BA 40); angular gyrus in the IPL (BA 39); pars opercularis and triangularis of the inferior frontal gyrus (BA 44/45); fusiform gyrus (BA 37); and lateral occipito-temporal cortex (BA 19). Additional areas hypothesized to be involved in spatial cognition, attention, and executive functions that may lead to higher IQ scores included the superior parietal lobule (BA 7), rostral middle (BA 46/9), and superior frontal cortices (BA 8/9); anterior cingulate (BA 24), and cuneus (BA 19). The program outputs a current estimate value for each voxel and each 4 ms time point. This value was then used to compute the dependent measure used in subsequent analyses namely the average current across all voxels defining each of the ROIs listed above and across all of the 4 ms time points comprising 13 successive 50 ms time bins (100–150, 150–200 ms, etc., up to 800 ms).

### ANALYSES

The overall model used in the group-level analyses was in the form of a RD group (RD vs. typical) by IQ group (higher vs. lower) ANCOVA with age as a covariate. Significant interactions were followed by one-way, simple main effects tests. The dependent measure in each analysis was averaged degree of activity for each ROI and 50-ms time window starting at 100 ms post-stimulus onset and ending at 750 ms. A nominal alpha level of 0.0038 was used to correct for family wise type I error rate.

Associations between degree of activity and achievement/IQ measures were then explored for those ROIs and time windows where significant main effects or interactions were found in the ANCOVAs. In addition, correlations between degree of activity in BA 37/19 with reading achievement and IQ were conducted as planned analyses, given that in our previous report (Simos et al., 2011) significant associations were found with reading achievement despite lack of significant differences between RD Groups in these regions. For ROIs and time windows where significant correlations with reading achievement scores were found, we tested the regulating role of IQ through moderated regression models conducted with SPSS macros developed by Hayes (model 2; Hayes, 2013). In this model the following equation was used to estimate reading achievement scores:

$$Y=i_{y}+c^{\prime }X+b_{1}M+b_{2}W+b_{3}X_{*}M+b_{4}X_{*}W+e_{y},$$

where *Y* represents WJ-WA or WJ-Letter Word Identification subtest standard scores, *X* represents degree of activity in a particular ROI and time bin, and *M* and *W* indicate the moderator variables (VIQ and PIQ, respectively). All variables were centered to their respective grand means before entered into the analyses.

## RESULTS

### GROUP-LEVEL ANALYSES

**FIGURE 1:**
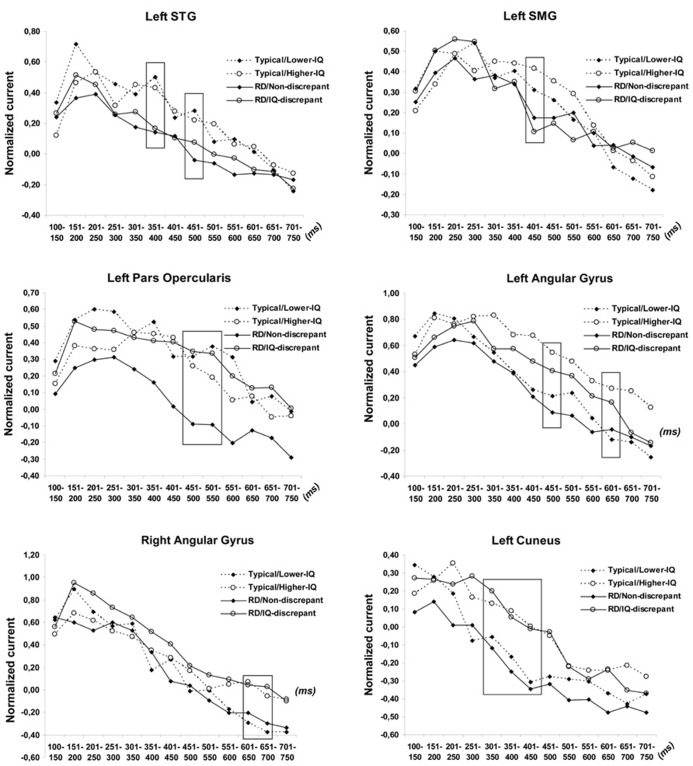
**Time course of estimated neurophysiological activity (nano-Amperes normalized to baseline) associated with pseudoword reading in the superior temporal (STG), supramarginal (SMG), angular gyri, and cuneus for each of the four group of participants.** Stimulus onset is at 0 ms. Time windows of significant group differences are marked by squares.

ANCOVA results revealed significant interactions of RD Group and IQ Group for left pars opercularis activity at 500–550 [*F*(1,122) = 9.11, *p* < 0.003] and at 550–600 ms post-stimulus onset [*F*(1,122) = 11.22, *p* < 0.001; see **Figure 1**]. Follow up tests showed that RD students who met the IQ-discrepancy criterion had higher degree of activity than Lower-IQ RD students at both time windows [*F*(1,62) = 8.64, *p* < 0.003 and *F*(1,62) = 10.99, *p* < 0.002, respectively]. Moreover, lower degree of activity in this region was found for poor as compared to typical readers of overall lower, yet comparable IQ [Lower-IQ RD students vs. Lower-IQ typical readers: *F*(1,51) = 11.21, *p* < 0.002 and *F*(1,51) = 15.04, *p* < 0.0001, respectively]. Higher-IQ RD and Higher-IQ typical readers demonstrated comparable degree of activity (*p* > 0.3). There were no other significant two-way interactions.

There were, however, significant main effects of IQ Group in three regions, indicating higher degree of activity for Higher-IQ than for Lower-IQ students: the left angular gyrus [between 450–500, *F*(1,122) = 8.52, *p* < 0.003, and 600–650 ms, *F*(1,122) = 8.22, *p* < 0.0037], the right angular gyrus [between 600 and 700 ms, *F*(1,122) = 8.84, *p* < 0.003 and *F*(1,122) = 9.30, *p* < 0.003] and the left cuneus [between 300 and 450 ms, *F*(1,122) = 10.97, *p* < 0.001, *F*(1,122) = 8.44, *p* < 0.003, and *F*(1,122) = 8.74, *p* < 0.003].

Finally, as expected based on our previous results (Simos et al., 2011) average activity in the left STG [between 350–400, *F*(1,122) = 11.25, *p* < 0.001 and 450–500 ms, *F*(1,122) = 11.83, *p* < 0.001] and left SMG [between 400 and 450 ms, *F*(1,122) = 8.15, *p* < 0.003] was stronger among typical than RD students, independent of IQ, as indicated by main effects of RD Group.

### BRAIN-ACHIEVEMENT/IQ ASSOCIATIONS

Correlational analyses conducted on the entire sample (*n* = 127) indicated that degree of activity in the left pars opercularis was positively associated with VIQ (*r* = 0.21 at 400–450 ms, controlling for PIQ and reading achievement) but not with PIQ or reading achievement (*r* < 0.1). Conversely, degree of activity in the left angular gyrus (*r* = 0.24 at 450–500 ms) and cuneus (*r* = 0.30 at 300–350 ms and *r* = 0.28 at 450–500 ms) was positively associated with PIQ (controlling for VIQ and reading achievement) but not with VIQ or reading achievement (*r* < 0.2). These correlations account for small amounts of the association of IQ and brain function.

WJ-WA scores were positively associated with left STG activity (*r* = 0.31 at 350–400 ms) as reported previously in a partially overlapping sample (Simos et al., 2011). Although activity in the left fusiform gyrus did not vary as a function of RD- or IQ-group, planned correlational analyses replicated the significant positive association between left fusiform activity at 200–250 ms and performance on the WJ-WA task (*r* = 0.37, *p* = 0.003), which was restricted to typical readers (*r* = -0.08 in the entire RD group). Correlations between left fusiform activity and IQ scores was <0.15 across all time windows.

### MODERATED REGRESSION ANALYSES

A stronger test of the role of IQ is the moderated regression model of the association between (a) left STG activity at 350–400 ms and (b) left fusiform activity at 200–250 ms and WJ-WA scores in the entire sample, including VIQ and PIQ measures as moderators. This analysis did not show that different IQ scores served as significant moderating effects (*p* > 0.2).

## DISCUSSION

The present study assessed whether reading skill-related variability in the degree of regional cortical activity varied as a function of participant verbal or PIQ. Our findings largely replicated the earlier work of Tanaka et al. (2011) using assessments of IQ that included measures of fluid and crystallized intelligence and a composite that represented a more traditional index than a receptive vocabulary measure. In addition, our higher-IQ RD subgroup met a formal IQ-discrepancy standard. As hypothesized, the most consistently found signs of aberrant brain function in RD, involving the left temporo-parietal cortex (e.g., Pugh et al., 2000; Joseph et al., 2001; Jobard et al., 2003) were not moderated by IQ. There was one exception to this trend involving the left pars opercularis, where hypoactivation in RD vs. typical readers depended upon IQ: this effect was observed only among lower-IQ participants. Across participants, degree of activity in this region was positively related to VIQ but did not correlate with reading skill. This finding may help account for discrepancies across previous neuroimaging studies with respect to reading skill-related effects on the degree of left inferior frontal gyrus activation. Some studies have reported increased activation among RD students and attributed this finding to compensatory processes (Shaywitz et al., 2002; Cao et al., 2006), whereas others failed to find group differences (Temple et al., 2001; Maisog et al., 2008; Simos et al., 2011). Increased activation depending on the type of comparison has been reported in another study (RD > age-matched controls whereas no difference was noted between RD and reading-matched controls; Hoeft et al., 2007a). There have also been reports of group differences in the opposite direction (Richards et al., 2002; Simos et al., 2007). The present results highlight a potential confounding role of VIQ in these earlier reports which is discussed further below in the context of the two main topics addressed by the present study, namely associations between brain activation, individual reading ability, and IQ.

### FUNCTIONAL BRAIN CORRELATES OF GENERAL COGNITIVE ABILITY

We identified three left hemisphere sites where degree of activity varied with general cognitive ability: the angular gyrus, pars opercularis, and cuneus. Correlational analyses suggested that activity in the angular gyrus and cuneus was more closely linked to PIQ, whereas a positive association was found between pars opercularis activation and VIQ. Although small but significant, these correlations are not surprising given the rising body of neuroimaging evidence regarding associations of morphometric and/or hemodynamic measures with IQ in children and adolescents. These include correlations between fractional anisotropy (Tamnes et al., 2010), cortical thickness (Kamara et al., 2009), gray matter volume (Taki et al., 2012), and local efficiency indices (derived from resting-state fMRI data; Wu et al., 2013) in inferior parietal and/or inferior frontal cortices with IQ.

Determining the nature of brain-IQ associations is rendered exceedingly difficult, in view of the multitude of available, complementary anatomical, and physiological measures of brain integrity and of the multidimensional nature of IQ tests and component cognitive processes each IQ task involves. A promising approach, that is currently explored by our group, involves first computing measures of regional interdependence between MEG-derived cortical activation time series, serving to establish real-time functional connectivity profiles during task performance. Specific elements of these profiles, along with more traditional measures of the degree of regional brain activity, may then be linked to particular reading-related processes (such as phonological decoding or word recognition). Anatomic features of the purported functional network (e.g., cortical thickness of implicated regions and anisotropy of underlying white matter) may then be used to refine specific network elements (see for example Hoeft et al., 2007b).

A further issue that deserves consideration is the potential confounding effect of age in the associations between IQ, reading achievement and brain activity. This possibility is raised by evidence that heritability of IQ varies with age (Brant et al., 2013), as does the anatomical distribution of associations between IQ and cortical thickness (Shaw et al., 2006).

### CORTICAL REGIONS INVOLVED IN PHONOLOGICAL DECODING

Two sites were identified for showing neurophysiological activity that varied as a function of phonological decoding ability, as measured outside the scanner through standardized tests: the superior temporal and fusiform gyri in the left hemisphere. Further, at the group level, RD students showed reduced degree of activity in the left supramarginal gyrus compared to typical readers. Similar findings have been reported by several studies (Cao et al., 2006; Hoeft et al., 2007a; Maisog et al., 2008; van der Mark et al., 2009; Simos et al., 2011; Tanaka et al., 2011), and is generally interpreted as highlighting the key role of cortex in the temporo-parietal junction for sub-word level phonological processing and analysis (Beauvois and Derouesne, 1979; Caplan et al., 1995; Scott et al., 2000; Specht et al., 2003; Jacquemot et al., 2003; Majerus et al., 2005).

Another region that is often implicated in reading and RD is the angular gyrus (in the IPL). In the present study, we found associations between IQ and activity in this region, independently of individual reading ability which, however, do not preclude its regular involvement in the reading process, given that these were observed during performance of a reading task. Moreover, a role of the IPL within the brain network for reading has been established by lesion (Philipose et al., 2007), electrocortical stimulation (Roux et al., 2004), and imaging studies (Booth et al., 2003). Nevertheless, reports implicating the left IPL in the pathophysiology of RD are less consistent across studies (see for instance Eden and Zeffiro, 1998; Pugh et al., 2000; Temple et al., 2001; Simos et al., 2007). In addition, this area of the brain is involved in a wide range of linguistic and non-linguistic cognitive processing reflecting its role as a cross-modal association area.

With respect to activity in the fusiform gyrus, the positive association between degree of activation and individual differences in phonological decoding ability is in accordance with previous neuromagnetic (Simos et al., 2011) and hemodynamic studies (Shaywitz et al., 2003). It is widely postulated that the role of ventral occipitotemporal cortices in phonological decoding tasks (and reading in general) concerns processing and storage of orthographic information (graphemic patterns; Tarkiainen et al., 1999; McCandliss et al., 2003; Philipose et al., 2007).

Reading disability group differences in left inferior frontal activation have been quite variable in both degree and direction with some studies reporting increased activation in RD (Shaywitz et al., 2002; Cao et al., 2006) and others reduced activation (Richards et al., 2002; Simos et al., 2007). Such inconsistencies and our failure to establish a significant (across or within RD groups) association between degree of left pars opercularis activity and decoding skill raise questions regarding the role of this region within the brain network responsible for phonological decoding. Our finding that only lower-IQ RD students demonstrated decreased pars opercularis activity as compared to typical readers of comparable IQ, may suggest that deficient engagement of this region for decoding in struggling readers depends upon cognitive ability and especially general language ability. It has been proposed that increased activity in this region in RD indicates increased “neural effort” in order to cope with the higher level of difficulty imposed by the decoding task (compared to the level of difficulty experienced by typically achieving readers; e.g., Hoeft et al., 2007a). Current results suggest a link between pars opercularis activation and verbal functions (as indicated by a significant correlation with VIQ in the entire sample). Failure to demonstrate this compensatory neural response by lower-IQ RD students may, therefore, indicate reduced capacity to engage in compensatory strategies for decoding. Such strategies may include articulatory recoding and/or access to stored real-word phonological representations. Moreover, recruitment of such compensatory strategies would pose greater demands upon working memory, as compared to the application of a straightforward, well-learned phonological conversion routine. Thus, lack of inferior frontal hyperactivity is also consistent with the key role of working memory in cognitive control and IQ (Conway et al., 2003; Wiley et al., 2011).

In sum, the present results largely confirm and extend the earlier report of Tanaka et al. (2011). A novel finding that may have implications for future imaging studies of RD concerns the potential confounding role of VIQ in assessing reading achievement group differences on inferior frontal activation. However, the fact that this relation was with VIQ and not PIQ is consistent with other findings suggesting that lower IQ children with RD are more impaired in overall language ability (Morris et al., 1998). Additionally, positive correlations between degree of activity and IQ were found in three cortical regions – the angular gyrus and cuneus (with PIQ) and the pars opercularis (with VIQ) – in the context of a task that did not require, at least in principle, complex cognitive operations such as those tapped by mainstream IQ tests. Given that degree of activity in these regions did not correlate with reading achievement, we may surmise that they play an auxiliary role in decoding. Moreover, the temporal resolution of magnetoencephalography made it possible to determine the time windows during which neurophysiological activity correlated with reading achievement or IQ. In all cases these effects reflected activity taking place later than 200–250 ms after stimulus onset (i.e., the time window when the degree of activity in the left fusiform gyrus correlated with reading achievement). Although circumstantial, this evidence is consistent with the notion that significant activations in the angular gyrus, cuneus, and IPL were involved in a post-orthographic processing stage.

To conclude, there is not strong evidence of a need to use an IQ-discrepancy criterion in neuroimaging studies. Although there were some differences between higher and lower IQ groups in the inferior frontal and angular gyrus regions, these seem to be related to overall language proficiency and the difference in VIQ – a selection criterion – and not to reading, where the IQ groups did not differ. In addition, so long as the lower threshold is set at a level not associated with intellectual disabilities (typically above the second percentile), the range of IQ scores in the final sample does not seem critical. In fact, because the population of all children with RD has lower IQ scores than typically achieving children, matching on IQ or setting the lower threshold too close to average may result in an unrepresentative sample. In behavioral studies, there is little evidence that IQ is strongly related to prognosis, intervention response, or cognitive skills related to reading (Fletcher et al., 2007). As in the present study, there are differences in cognitive skills not related to reading. Moreover, the IQ-achievement discrepancy is no longer required in the US Individuals with Disabilities in Education Act and was abandoned by DSM-5. The reductions in VIQ associated with RD are just as likely caused by language weaknesses also associated with RD or result from RD and the impact of poor reading on vocabulary and other forms of language learning. Focusing on relations of RD with more discrete skills than those typically measured by IQ tests would seem an important direction for neuroimaging research. In addition, further exploration attempting to differentiate the role of the inferior frontal and angular gyrus regions in language vs. reading skills would be indicated.

## Conflict of Interest Statement

The authors declare that the research was conducted in the absence of any commercial or financial relationships that could be construed as a potential conflict of interest.
